# Prolonged Exposure to Antiretroviral Therapy and Risk of Developing Hypertension Among HIV-Infected Clinic Attendees: A Pilot Study in Rural Eastern Cape Province, South Africa

**DOI:** 10.3390/ijerph22091397

**Published:** 2025-09-07

**Authors:** Teke Apalata, Urgent Tsuro, Olufunmilayo Olukemi Akapo

**Affiliations:** 1 Department of Laboratory Medicine and Pathology, Faculty of Medicine and Health Sciences, Walter Sisulu University, Mthatha B-5110, South Africa; oakapo@wsu.ac.za; 2 National Health Laboratory Service, Nelson Mandela Academic Hospital, Mthatha B-5110, South Africa; 3 Division of Public Health, Department of Community Medicine, Faculty of Health Sciences, Walter Sisulu University, Mthatha B-5110, South Africa; tsurourgent@gmail.com

**Keywords:** ART, HIV, age, triglyceride levels, hypertension

## Abstract

Antiretroviral therapy (ART) has significantly improved outcomes in individuals with human immunodeficiency virus (HIV), yet its long-term cardiovascular effects, especially on hypertension risk, remain debated. This pilot study investigated hypertension risk factors in HIV-positive patients undergoing ART and aimed at hypothesis generation rather than drawing definitive causal conclusions. Seventy HIV-infected adults without baseline hypertension were enrolled and followed. Hypertension was defined using the 2017 ACC/AHA guidelines by the South African Hypertension Society. Data on demographic, anthropometric, metabolic, inflammatory, coagulation, and HIV-related variables were collected. Cox regression analysis identified independent predictors of hypertension. Participants had a median age of 37 years (IOR = 10.96), with 84.3% being female. After a median ART exposure of 61.01 months (range: 2–164), 27 individuals (38.6%) developed high blood pressure. In multivariable Cox models adjusting for metabolic syndrome and BMI, age ≥ 35 years was associated with a 2.2-fold higher hypertension risk (Hazard Ratio [HR]: 2.2; 95% Confidence Interval [CI]: 1.04–4.55; *p* = 0.04). Elevated triglycerides significantly increased risk, with a 7.9-fold higher likelihood of hypertension (HR: 7.9; 95% CI: 1.04–59.5; *p* = 0.046). ART regimen type, whether initial or current, did not independently predict hypertension. In conclusion, hypertension is prevalent during ART. We hypothesized that traditional cardiovascular risk factors, notably age ≥35 years and hypertriglyceridemia, were key independent predictors, emphasizing the need for routine cardiovascular risk assessment in HIV management.

## 1. Introduction

According to the United Nations statistics, Eastern and Southern Africa possessed 20.6 million people living with the human immune-deficiency virus (HIV) in 2018, of which 13.8 million were accessing antiretroviral therapy (ART) [1]. Sub-Saharan Africa (SSA) bears the greatest load of the HIV crisis, making up approximately 54% of the global HIV burden [2]. The introduction of ART marked a very big improvement in the life expectancy of people living with HIV [3,4,5]. Hypertension remains a growing health concern globally, with an increase among people living with HIV (PLWH), particularly in low- and middle-income countries. Recent studies highlighted the complex interplay between HIV infection, antiretroviral therapy (ART), and traditional cardiovascular risk factors, underscoring the need for targeted research in underrepresented settings [6,7].

Aging-related diseases including hypertension begin to appear as life expectancy increases [8]. Due to urbanization and westernization, hypertension and other chronic non-communicable diseases (NCDs) are at a rise in SSA [9,10]. Research findings by [11] Nartey et al. (2023) in a study where causal association between ART and hypertension was studied using propensity score showed a prevalence of hypertension was significantly greater among individuals exposed to ART (42.4%) compared to individuals who were not exposed to ART (17.0%). Furthermore, systolic (12.0 mm Hg) and diastolic (6.1 mm Hg) blood pressure elevations were linked to ART usage [11]. ART-treated HIV-positive patients exhibit a much greater morbidity rate for cardiovascular disease (CVD) compared to non-ART-treated patients [12].

Studies have shown that there is a higher risk ratio (RR) with people living with hypertension (PWH) in North America and Europe than those living in Africa with increased incidence of hypertension among HIV-positive people receiving antiretroviral therapy in these regions [13]. Reports have shown that there is an association between elevated blood pressure in people with HIV and dolutegravir-based antiretroviral therapy regimens. There is tendency in men using dolutegravir to show an elevated risk of hypertension [14]. The recent increase in CVD morbidity and mortality may be an indicator of HIV-infection-related risk factors, ART metabolic effects, and chronic inflammation driven by the virus [15]. Recent regional and global studies present a high and growing incidence of hypertension, with low rates of verification, treatment, and management in sub-Saharan Africa (SSA) [16]. A study by [17] S reported that approximately a third (32%) of the people in a study assessing hypertension a year after receiving ART acquired the condition. A higher risk was linked to the use of stavudine and non-nucleoside reverse transcriptase inhibitors (NNRTIs), especially efavirenz. Additionally, a higher risk of hypertension has been associated with conventional characteristics such as advanced age and a higher body mass index (BMI) [17,18]. Prior to the use of ART, hypertension was less prevalent in people with HIV [19,20]. Obesity and older age are increasingly becoming associated with HIV infection as well [21,22]. A 2024 meta-analysis and systematic review reported that overall prevalence of hypertension remained 21.9%; this study evaluated data from 48 studies comprising 193,843 PLWH. Each individual in this study had a CD4 count of at least 200, and prevalence rates were higher in men and those on ART [23]. Ref. [23] investigated the incidence and risk factors of metabolic syndrome (MetS) in people with HIV who were using ART in Ethiopia between 2022 and 2023. The results of the study revealed that a longer ART duration was linked to a higher risk of metabolic problems. Particularly, the prevalence of MetS was 37.6%, and each participant’s average length of time on combination ART was 10 years. Age over 45, female sex, body mass index (BMI) greater than 25 kg/m^2^, and usage of lopinavir/ritonavir or efavirenz-based ART regimens have been identified as independent risk factors [24]. Another study that was conducted in Kenya noted that there is a high prevalence of obesity and hypertension among HIV-positive people; this high prevalence varied between women and men, however. A systematic review conducted in sub-Saharan Africa by [22] established that the occurrence of lower BP was more prevalent among HIV-positive adults than HIV-negative adults [23,25]. This is supported by a population-based study conducted in South Africa amongst HIV-positive individuals, which pointed out that hypertension was not common among HIV-positive patients [26].

The long-term use of ART in HIV-infected patients results in obesity, immune complications, and drug toxicity [27]. Although there have been recent studies that investigated the prevalence of hypertension and its associated risk factors in HIV-infected people on ART in SSA, there is an urgent need for further research focusing on how HIV and hypertension combine in sub-Saharan Africa, given the region’s high prevalence of both diseases [28,29]. This study focuses on hypertension risk among PLWH in a rural South Africa context, where access to healthcare is often limited and where the dual burden of infectious and non-communicable diseases places additional strain on health systems. By incorporating both anthropometric and metabolic markers, this approach aligns with current public health priorities aimed at integrating chronic disease management within HIV care, especially in resource-constrained environments [30,31].

We conducted this pilot and exploratory study to evaluate the prevalence of hypertension, its risk factors, and generate hypotheses rather than drawing definitive causal conclusions in a cohort of HIV-positive clinic attendees on ART in a rural area of the Eastern Cape province in South Africa, considering the scarcity of data on CVD risk factors in the context of HIV in our setting.

## 2. Materials and Methods

We conducted a pilot study among 70 HIV-infected patients receiving ART attending Ngangelizwe community health centre (NCHC), a government healthcare facility, located at King Sabata Dalindyebo (KSD) local municipality in South Africa’s rural Eastern Cape province between 1 February and 28 February 2019 and there was no loss to follow-up. This pilot study was conducted on a limited number of participants (n = 70) to allow for a strict follow-up mechanism, preventing the occurrence of missing data. The proportional hazards assumption was tested using Schoenfeld residuals and log-log survival plots, and no significant violations were observed. Written informed consent was obtained from the patients. Patients were consecutively enrolled into the study if they were aged 18 years and above and diagnosed with HIV infection but free from hypertension at the initiation of ART (the regimens of ART used are shown in Table 1). The World Health Organization (WHO) STEPwise instrument for data collection and measurement of non-communicable diseases (NCD) risk factors was used [32,33]. Demographic data, medical history (including history of type 2 diabetes mellitus (T2DM), hypertension, tobacco smoking, and alcohol use), and physical examination findings were recorded using the WHO STEPwise tool as shown in Table 2 and Table 3. Blood pressure was measured using a calibrated automated sphygmomanometer following the WHO STEPS protocol. Each participant’s BP was measured three times at 2-min intervals in a seated position after 5 min of rest, and the average of the three readings was used in the analysis. Hypertension was defined as SBP ≥ 140 mmHg and/or DBP ≥ 90 mmHg or self-reported use of antihypertensive medication, in line with South African hypertension guidelines. Abnormal BP refers to any single elevated reading during baseline screening but not meeting the criteria for a confirmed diagnosis of hypertension. Weight and height were measured as shown in Table 2. Body mass index (BMI) was defined as underweight (BMI < 18.5 kg/m^2^), normal (BMI: 18.5 to 24.9 kg/m^2^), overweight (BMI: 25.0 to 29.9 kg/m^2^), and obese (BMI ≥ 30 kg/m^2^). The presence of metabolic syndrome (MetS) among the study participants was ascertained using criteria developed by the International Diabetes Federation (IDF) and the National Cholesterol Education Program (NCEP) Adult Treatment Panel (ATP) III [34,35]. According to IDF criteria, MS was characterized by the presence of central obesity (waist circumference ≥ 94 cm for men or ≥80 cm for women) together with two of the following diagnostic criteria: (1) elevated triglyceride levels (≥150 mg/dL or ≥1.7 mmol/L); (2) low HDL cholesterol (<40 mg/dL or <1.04 mmol/L in men and <50 mg/dL or <1.29 mmol/L in women); (3) hypertension (BP ≥ 140/≥90 mmHg); and (4) fasting hyperglycemia (glucose level ≥ 5.6 mmol/L or ≥100 mg/dL) or previous diagnosis of diabetes. The waist circumference was measured at the belly button using a tailor’s tape. Random blood glucose, glycated hemoglobin (HbA1c), lipid profiles including total cholesterol (TC), low-density lipoprotein (LDL), high-density lipoprotein (HDL), and triglycerides, D-dimmer, and C-reactive protein (CRP) were measured on the Cobas 6000 (Roche, Germany). CD4+ T-cell counts were measured using Partec CyFlow Counter (Cyflow SL, Partec, Munster, Germany), whereas HIV and Hepatitis B measurements were performed using the Multiplex HIV, HCV & HBV nucleic acid test for use on the Cobas^®^ 6800/8800 Systems (Roche, Germany) [36,37]. For HIV testing, at the clinic, a serial algorithm with Determine Rapid HIV-1/2 antibody (Abbott Laboratories, Abbott Park, IL, USA) followed by Unigold Rapid HIV Test (Trinity Biotech, PLC, IDA Business Park, Bray, County Wicklow, Ireland) was used. Cobas m511 integrated hematology analyzer (Roche, Basel, Switzerland) automatically prepared blood samples for immediate testing of platelet counts, erythrocyte sedimentation rate (ESR), and white cell counts. Continuous data were summarized as median (IQR) whilst categorical data were presented as proportions (%). The Chi-square test was used for testing relationships between variables of interest. If the outcome was a continuous variable, we used linear regression. Since the study outcome was ‘time to event’, we used Cox proportional hazard models, with a *p*-value < 0.05 being significant. IBM SPSS v.23 (Chicago, IL, USA) was used for statistical data analysis.

## 3. Results

Of the 70 HIV-infected patients, 27 (38.6%) developed abnormal BP, 13/27 (48.10%) within the initial 5 years of ART exposure; 10 (37.04%) between 5 and 10 years of ART exposure; and the remaining 4 (14.81%) developed abnormal BP after 10 years of ART exposure.

**Figure 1:**
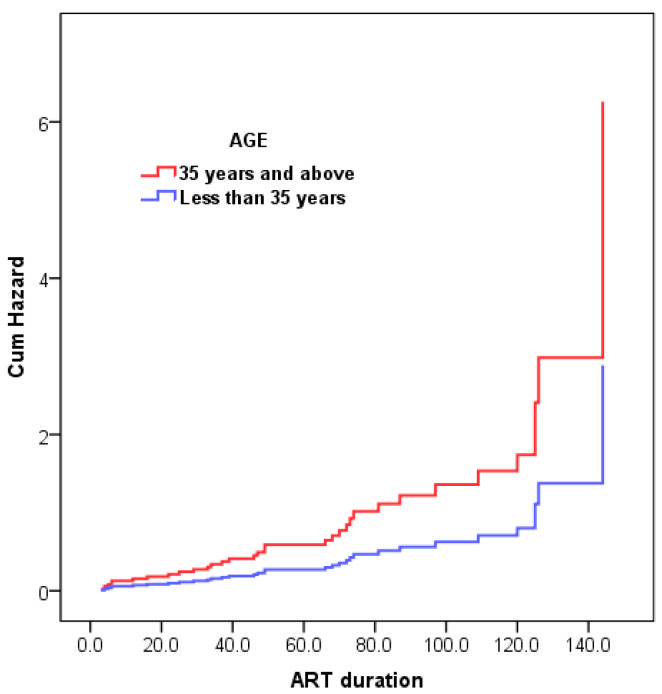
Cumulative hazard of event by age group over duration of ART.

**Figure 2:**
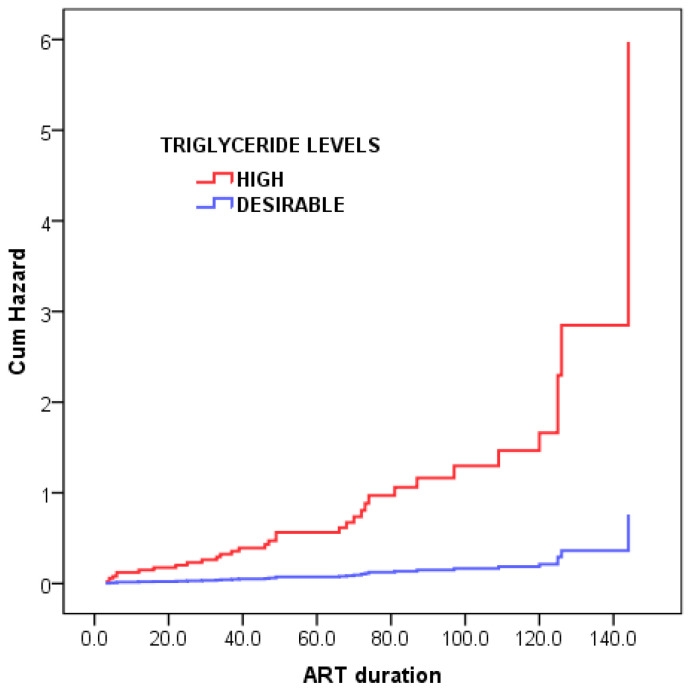
Hypertriglyceridemia as an independent risk factor for the development of abnormal BP over the course of ART in HIV-infected patients (n = 70).

Table 2 displays patients’ characteristics as well as factors associated with the development of high BP following univariate/bivariate analysis. Table 2, Table 3 and Table 4 showed the associations between selected variables of interest and the occurrence of hypertension among HIV positive patients during ART. Univariate/bivariate analyses (Table 2 and Table 3) showed that patients aged ≥35 years, overweight or obese, with high blood levels of triglyceride, and diagnosed with MS using NCEP criteria. Figure 1 and Figure 2 shown that multivariable Cox models adjusting for metabolic syndrome and BMI, age ≥ 35 years was associated with a 2.2-fold higher hypertension risk (Hazard Ratio [HR]: 2.2; 95% Confidence Interval [CI]: 1.04–4.55; *p* = 0.04). Elevated triglycerides significantly increased risk, with a 7.9-fold higher likelihood of hypertension (HR: 7.9; 95% CI: 1.04–59.5; *p* = 0.046).

Figure 1 shows that older individuals (≥35 years) have a higher cumulative risk of the event than younger individuals, particularly as ART duration lengthens.

Figure 2 above presents over the course of ART 2 times (Hazard Ratio: 2.2; 95% CI: 1.04–4.55; *p* = 0.04) and 8 times (Hazard Ratio: 7.9; 95% CI: 1.04–59.5; *p* = 0.046) higher likelihood of developing hypertension, respectively. Variables in the Cox proportional hazards model are displayed.

## 4. Discussion

Studies have shown that high levels of triglyceride contribute to the thickening of the artery walls (arteriosclerosis), resulting in increased risk of hypertension, stroke, heart attack, and other cardiovascular diseases. In the present study, we expanded our comparison to include findings from similar African cohorts, particularly those conducted in rural and peri-urban populations in East and Southern Africa. Our findings align with previous studies from East Africa, such as research from rural Uganda and Kenya, which reported comparable rates of hypertension among PLWH, underscoring the growing burden of non-communicable diseases (NCDs) in these traditionally underserved settings [38,39]. Similarly, data from rural South Africa have highlighted a rising prevalence of hypertension in HIV-positive populations, driven by a combination of long-term ART exposure, aging, and the increasing prevalence of traditional risk factors such as obesity and dyslipidemia [40]. Most patients in this study were either obese or overweight, consistent with findings by [40] that weight reduction through dietary interventions improves BMI and enhances glucose and lipid metabolism, including lowering triglyceride levels. This supports the recommendation for weight loss to reduce excess calories that contribute to elevated triglycerides [41]. Furthermore, most of the patients in this study who had high blood pressure were drinking alcohol; however, this was not statistically significant. While alcohol was not significantly associated with the outcome in our analysis, this finding should be interpreted with caution and does not preclude a possible association in larger or more diverse populations. The latter is high in calories and sugar, and previous studies have demonstrated that regular consumption of alcohol had a potent effect on levels of triglycerides [42]. It is evident from this study that traditional risk factors (not HIV- and ART-related factors) were key players in the development of hypertension among HIV-infected patients on chronic medication; therefore, policies on healthy eating habits should be promoted. The use of healthier fats such as fat found in plants, vegetables, and fish, which are high in omega-3, should be recommended. Regular exercise along with reducing sugar and processed carbohydrates are additional healthy lifestyle choices that patients can make to prevent elevated blood triglyceride levels [43].

Study has established a link between age and triglyceride levels. Triglyceride levels in men’s blood have been found to progressively rise until the age of 50, at which point it begins to lightly decrease. Nonetheless, as women age, their triglyceride levels continue to rise, mild hypertriglyceridemia is reported in women from the age of 60 years and 30 years in men [42]. Although age influences blood levels of triglycerides, it has been previously reported as being an independent risk factor for hypertension [44]. Prior research suggests that older adults typically have much higher blood pressure, but not everyone will experience this age-related rise in blood pressure, regardless of their HIV status or ART regimen [45]. Early identification of elevated blood pressure and metabolic abnormalities among people living with HIV (PWH) on ART highlights the need to integrate routine cardiovascular risk screening, including BMI, waist circumference, and lipid profiles, into existing ART monitoring protocols. This could improve early detection and management of hypertension and reduce long-term morbidity. Our findings in this study report traditional risk factors such as being older than 35 and having an elevated triglyceride, which suggest significant, independent, and highly associated risk factors in hypertension. The incidence of hypertension is also high among HIV-infected patients receiving chronic ART medication in this study. We acknowledge that this finding could be attributed to several factors: the relatively small sample size, which may have limited the statistical power to detect significant associations, the homogeneity of ART regimens among participants, with a large proportion receiving similar fixed-dose combinations, and the possibility of a true absence of effect in this specific population. We consider this result as clinically significant since the magnitude of effect is meaningful in practice, regardless of whether 95% CIs are wider; hence, we plan a further study with a large sample size and appropriate controls, including HIV-negative patients.

The limitations in this study include the pilot study not being powered to make definitive causal inferences due to a small sample size. It is, however, exploratory in nature for generating hypotheses. Although the proportional hazards assumption holds, we did not perform the Correction for Multiple Comparisons (FDR and Bonferroni). The pilot study was also conducted in one community health center only, limiting the possibility of generalization of our results There is need for future research with larger, more diverse cohorts and which employs longitudinal or randomized designs to better isolate regimen effects to be used.

## 5. Conclusions

The observed patterns of elevated blood pressure and metabolic abnormalities among people living with HIV on ART suggest a potential need to explore the integration of routine cardiovascular risk screening into existing ART monitoring protocols. While this pilot study is not powered to make definitive causal inferences, the preliminary findings underscore the value of further research. These exploratory results may provide a foundation for future studies that could support broader efforts to align HIV care with national strategies for managing HIV–NCD comorbidities.

There is a need for robust and expanded study throughout the province with comparable data among various settings to establish the findings from this study.

## 6. Future Research

Future research showing the sample size calculation of a new study that age-matches general population, taking into account the HIV-negative control group, will be considered.

The pilot study aimed at generating hypotheses for further research, which should have a sample size that allows for drawing conclusions. The sample size of the further study must allow for the following:

Formula (Schoenfeld Method)

To estimate the required number of events (n) to achieve 80% power at a 5% significance level in a Cox proportional hazards model, Schoenfeld formula will be used:

n = [(Z_1−_α/2 + Z_1−_β)^2^]/[p(1 − p) × (log(HR))^2^] (1)

where

- n: Number of events required;
- Z_1−_α/2: Z-value for significance level (e.g., 1.96 for 5%);
- Z_1−_β: Z-value for desired power (e.g., 0.84 for 80%);
- HR: Hazard Ratio to detect;
- p: Proportion of individuals in the treatment/exposure group.

## Figures and Tables

**Table 1:** ART regimen coding system definitions.

Code	Components (Drugs)	Full Names	Drug Class
**1T3E**	Tenofovir + Lamivudine + Efavirenz	Tenofovir disoproxil fumarate (TDF) + Lamivudine (3TC) + Efavirenz (EFV)	NRTI + NRTI + NNRTI
**1TFE**	Tenofovir + Emtricitabine + Efavirenz	Tenofovir disoproxil fumarate (TDF) + Emtricitabine (FTC) + Efavirenz (EFV)	NRTI + NRTI + NNRTI
**1S3E**	Stavudine + Lamivudine + Efavirenz	Stavudine (d4T) + Lamivudine (3TC) + Efavirenz (EFV)	NRTI + NRTI + NNRTI
**1S3N**	Stavudine + Lamivudine + Nevirapine	Stavudine (d4T) + Lamivudine (3TC) + Nevirapine (NVP)	NRTI + NRTI + NNRTI
**1T3N**	Tenofovir + Lamivudine + Nevirapine	Tenofovir disoproxil fumarate (TDF) + Lamivudine (3TC) + Nevirapine (NVP)	NRTI + NRTI + NNRTI

**Table 2:** Characteristics of the study participants and univariate/bivariate analysis of factors associated with the occurrence of abnormal blood pressure among the HIV-positive clinic attendees. The table reveals the factors associated with high blood pressure in the study.

Variables of Interest	Total N (%)	Blood Pressure		Chi-Square	*p*-Value
		Normal	High		
**Non-modifiable risk factors:**					
Age, years				4.58	0.032
<35	32 (45.71%)	24/32 (75%)	8/32 (25%)		
≥35	38 (54.29%)	19/38 (50%)	19/38 (70.4%)		
Gender				2.29	0.130
Male	11 (15.70%)	9/11 (81.8%)	2/11 (18.2%)		
Female	59 (84.30%)	34/59 (57.6%)	25/59 (42.4%)		
**Modifiable risk factors:**					
Alcohol consumption					
Yes	26 (37.1%)	15/26 (57.7%)	11/26 (42.3%)	0.24	0.621
No	44 (62.9%)	28/44 (63.6%)	16/44 (36.%)		
Cigarette smoking				0.41	0.521
Yes	13 (18.6%)	9/13 (20.9%)	4/13 (30.8%)		
No	57 (81.4%)	34/57 (59.1%)	23/57 (40.4%)		
History of type 2 diabetes mellitus				0.16	0.686
Yes	4 (6.2%)	3/4 (75.0%)	1/4 (25.0%)		
No	61 (93.8%)	39/61 (63.9%)	22/61 (36.1%)		
Body mass index				9.74	0.008
Normal	29 (41.4%)	24/29 (82.8%)	5/29 (17.2%)		
Overweight	20 (28.6%)	10/20 (50.0%)	10/20 (50%)		
Obesity	21 (30%)	9/21 (42.9%)	12/21 (57.1%)		

**Table 3:** Univariate and bivariate analysis of lipid profiles, glucose levels, coagulation and inflammatory markers, metabolic syndrome, HIV parameters, and ART regimens associated with hypertension among HIV-positive clinic attendees.

Variables of Interest	Total N (%)	Blood Pressure		Chi-Square	*p*-Value
		Normal	High		
**Lipid profile:**					
Total cholesterol, mmol/L				2.32	0.128
Desirable	60 (93.8%)	38/60 (63.3%)	22/60 (36.7%)		
High	4 (6.3%)	1/4 (25.0%)	3/4 (75%)		
LDL, mmol/L				1.60	0.205
Desirable	45 (97.8%)	28/45 (62.2%)	17/45 (37.8)%		
High	1 (2.2%)	0/1 (0%)	1/1 (100%)		
HDL, mmol/L				1.44	0.230
Desirable	45 (70.3%)	26/45 (57.8%)	19/45 (42.2%)		
Low	19 (29.7%)	14/19 (35%)	5/19 (20.8%)		
Triglyceride, mmol/L				13.15	0.0002
Desirable	46 (82.1%)	33/46 (77.1%)	13/46 (28.3%)		
High	10 (17.9%)	1/10 (10.0%)	9/10 (90.0%)		
Ratio total cholesterol/HDL				2.83	0.024
<4	56 (88.9%)	36/56 (64.3%)	20/56 (83.3%)		
4–6	6 (9.5%)	2/6 (33.3%)	4/6 (66.7%)		
>6	1/1 (1.6%)	1/1 (100%)	0/1 (0%)		
**Blood glucose tests**					
Random blood glucose, mmol/L				0.64	0.424
Normal	69 (98.6%)	42/69 (60.9%)	27/69 (39.1%)		
Abnormal	1 (1.4%)	1/1 (100%)	0/1 (0%)		
Glycated Hb, mmol/L				3.40	0.065
Normal	48 (68.6%)	26/48 (60.5%)	22/48 (81.5%)		
Abnormal	22 (31.4%)	17/22 (77.3%)	5/22 (22.72%)		
**Coagulation disorders**					
D-dimmer				1.58	0.208
Positive	30 (46.2%)	16/30 (53.3%)	14/30 (46.6%)		
Negative	35 (53.8%)	24/35 (68.5%)	11/35 (31.4%)		
**Metabolic syndrome**					
MS by NCEP				3.07	0.080
Present	31 (44.3%)	15/31 (48.4%)	16/31 (51.6%)		
Absent	39 (55.7%)	28/39 (71.7%)	11/39 (28.2%)		
MS by IDF				1.58	0.209
Present	31 (44.3%)	16/31 (51.6%)	15/31 (48.4%)		
Absent	39 (55.7%)	27/39 (69.2%)	12/39 (30.8%)		
**HIV-associated risk factors**					
WHO staging at ART initiation				1.96	0.58
Stage 1	34 (49.3%)	20/34 (58.8%)	14/34 (41.2%)		
Stage 2	15 (21.7%)	8/15 (53.3%)	7/15 (46.7%)		
Stage 3	18 (26.1%)	12/18 (66.7%)	6/18 (33.3%)		
Stage 4	2 (2.9%)	2/2 (100%)	0/2 (0%)		
Current HIV viral load, copies/mL				1.00	0.32
Detectable	39 (58.2%)	22/39 (56.4%)	17/39 (43.6%)		
Undetectable	28 (41.8%)	20/28 (71.4%)	8/28 (28.6%)		
Level of immunosuppression				0.38	0.943
Severe	4 (6%)	3/4 (75.0%)	1/4 (25.0%)		
Advanced	10 (14.9%)	6/10 (60.0%)	4/10 (40.0%)		
Mild	7 (10.4%)	4/7 (57.1%)	3/7 (42.8%)		
Normal	46 (68.7%)	29/46 (63.0%)	17/46 (36.9%)		
**ART-associated risk factors**					
Initial ART regiments **				2.14	0.71
1T3E	25 (35.7%)	15/25 (60.0%)	10/25 (40.0%)		
1TFE	33 (47.1%)	20/33 (60.9%)	13/33 (39.4%)		
1S3E	3 (4.3%)	3/3 (100%)	0/3 (0%)		
1S3N	5 (7.1%)	3/5 (60.0%)	2/5 (40.0%)		
1T3N	4 (5.7%)	2/4 (50.0%)	2/4 (50.0%)		
Current ART regiments				6.02	0.019
1T3E	16 (23.2%)	10/16 (62.5%)	6/16 (37.5%)		
1TFE	48 (69.6%)	30/48 (62.5%)	18/48 (37.5%)		
1S3E	2 (2.9%)	2/2 (100.0%)	0/2 (0%)		
1S3N	1 (1.4%)	0/1 (0%)	1/1 (100.0%)		
1T3N	2 (2.9%)	0/2 (0%)	2/2 (100%)		
ART duration, years				0.20	0.91
<5 years	36 (51.4%)	23/36 (63.5%)	13/36 (36.1%)		
5–10 years	24 (34.3%)	14/24 (58.3%)	10/24 (41.7%)		
>10 years	10 (14.3%)	6/10 (60.0%)	4/10 (40.0%)		

**Table 4:** Multivariate Cox-regression analysis displaying independent risk factors for the development of hypertension over the duration of ART exposure.

Risk Factors	Hazard Ratio (HR) (95% CI for HR)	B-Coefficient	SE	Wald Chi-Square	*p*-Value
Age, Years	2.169 (1.035–4.546)	0.774	0.378	4.206	0.04 *
Triglyceride, mmol/L	7.855 (1.037–59.469)	2.061	1.033	3.982	0.046 *

## Data Availability

Data from this study will be made available from the corresponding author upon request.

## Abbreviations

The following abbreviations are used in this manuscript:

ACC	American College of Cardiology
AHA	American Heart Association
ART	Anti-retroviral therapy
ANOVA	Analysis of variance
BMI	Body mass index
CD4	Cluster of differentiation 4
CI	Confidence interval
CRP	C-reactive protein
DBP	Diastolic blood pressure
HDL	High-density lipoprotein
HIV	Human immunodeficiency virus
LDL	Low-density lipoprotein
NCD	Non-communicable diseases
NHLS	National Health Laboratory Services
SBP	Systolic blood pressure
SD	Standard deviation
SSA	Sub-Saharan Africa
TC	Total cholesterol
WHO	World Health Organization
NRTI	Nucleoside Reverse Transcriptase Inhibitor
NNRTI	Non-Nucleoside Reverse Transcriptase Inhibitor
TDT	Tenofovir Disoproxil Fumarate
3TC	Lamivudine
FTC	Emtricitabine
EFV	Efavirenz
NVP	Nevirapine
d4T	Stavudine
